# Influence of Fused Deposition Modelling Nozzle Temperature on the Rheology and Mechanical Properties of 3D Printed β-Tricalcium Phosphate (TCP)/Polylactic Acid (PLA) Composite

**DOI:** 10.3390/polym14061222

**Published:** 2022-03-17

**Authors:** Karim Elhattab, Sarit B. Bhaduri, Prabaha Sikder

**Affiliations:** 1 Department of Mechanical, Industrial & Manufacturing Engineering, The University of Toledo, Toledo, OH 43606, USA; sarit.bhaduri@utoledo.edu; 2 EEC Division, Directorate of Engineering, The National Science Foundation, Alexandria, VA 22314, USA; 3 Department of Mechanical Engineering, Cleveland State University, Cleveland, OH 44115, USA; p.sikder@csuohio.edu

**Keywords:** PLA, tricalcium phosphate, composites, fused deposition modeling, nozzle temperature, rheology, crystallinity, mechanical properties

## Abstract

The primary goal of this study is to develop and analyze 3D printed structures based on a well-known composite known as β-Tricalcium Phosphate (TCP)– polylactic acid (PLA). There are some interesting aspects of this study. First, we developed 3D printable TCP–PLA composite filaments in-house, with high reproducibility, by a one-step process method using a single screw extruder. Second, we explored the physicochemical properties of the developed TCP–PLA composite filaments. Third, we investigated the effect of an FDM-based nozzle temperature of 190 °C, 200 °C, 210 °C, and 220 °C on the composite’s crystallinity and rheological and mechanical properties. Results confirmed the successful development of constant-diameter TCP–PLA composite filaments with a homogeneous distribution of TCP particles in the PLA matrix. We observed that a higher nozzle temperature in the FDM process increased the crystallinity of the printed PLA and TCP–PLA structures. As a result, it also helped to enhance the mechanical properties of the printed structures. The rheological studies were performed in the same temperature range used in the actual FDM process, and results showed an improvement in rheological properties at higher nozzle temperatures. The bare polymer and the composite polymer-ceramic melts exhibited lower viscosity and less rigidity at higher nozzle temperatures, which resulted in enhancing the polymer melt flowability and interlayer bonding between the printed layers. Overall, our results confirmed that 3D printable TCP–PLA filaments could be made in-house, and optimization of the nozzle temperature is essential to developing 3D printed composite parts with favorable mechanical properties.

## 1. Introduction

Additive manufacturing (AM) has received significant attention in the biomedical field due to several advantages such as its ability to efficiently develop complex and customized structures, its ease in materials processing, its efficiency in material consumption, its elimination of post processing steps such as machining, and, hence, less manufacturing or production time and cost [1,2,3,4]. Fused deposition modeling (FDM) is one of the most used AM processes in the biomedical field because of its low cost and simple operation compared to the other AM processes [5,6]. Moreover, FDM can be utilized for a broad spectrum of biomaterials [6,7,8]. Polylactic acid (PLA) is one of the extensively studied biomaterials in the biomedical field [9,10,11], and it has been successfully used in many biomedical applications [12,13,14,15,16]. In particular, in orthopedics and dentistry [17,18], PLA became the material of choice. For instance, it was successfully used for pins, screws, washers, and darts [10]. Although PLA has good mechanical properties, biocompatibility, FDA-approved biodegradability and facile processability [12,19], its slow biodegradability and the acidic environment caused by lactic acid during the degradation of PLA limits the in vivo application of implantable PLA scaffolds.

Producing 3D printable PLA-based biocomposite can be a convenient solution to address the above-mentioned challenge. For instance, the ceramic component in a PLA-bioceramic composite will be able to neutralize the acidic microenvironment caused by the byproducts of PLA. Second, the 3D printable PLA-based composite filaments can be used in an FDM process to develop structures (e.g., implants) with precise pore size and geometry and minimize material involvement in the implant [20]. Third, a biocomposite produced by melt mixing will avoid the use of toxic solvents as opposed to conventional methods of scaffold fabrications [21,22]. It is crucial to avoid using solvents in fabricating biocomposites because solvent residue has a poisonous effect in vitro and in vivo [23]. Thus, a 3D printable PLA-bioceramic filament composition could be a favorable solution to develop biocompatible implants that would create a less acidic microenvironment.

Calcium phosphate is a well-known bioceramic in orthopedics as 65% of the bone composition is hydroxyapatite (HA). In particular, β-tricalcium phosphate (TCP) has excelled on HA and other calcium phosphate phases for two main reasons. First, its degradation rate is three times faster than that of HA [22]. Second, it is completely absorbable. Another excellent property is that it has the ability to buffer an acidic environment [24]. The addition of TCP component within the PLA matrix improves the in vitro degradation and biocompatibility of the TCP–PLA composite by increasing the dissolution rate of PLA and also buffering the acidic environment by releasing the calcium phosphate particles [25,26,27]. Many studies in the literature studied the influence of TCP on biological and 3D printing performances when it is added as a bioceramic component into a polymer matrix. Backes et al. studied the effect of TCP content in PLA matrix in developing 3D printable biocomposite [28]. In this study, TCP–PLA biocomposite was fabricated with different content of TCP 5, 10, and 25 wt% by melt compounding. The results revealed excellent bioactivity of TCP–PLA composites as carbonated hydroxyapatite was formed on the composites with different TCP content when they were immersed in simulated body fluid (SBF) solution for 7 and 21 days. The biocompatibility assay showed an increase in cell viability of the composite with 10 wt% TCP compared to the control and the 5 wt% TCP composite. The composites with 5 and 10 wt% experienced good rheological properties similar to the pure PLA, and they were used to 3D print bone ligament fixation screws. However, the composite with 25 wt% TCP experienced a large agglomeration of TCP particles, significantly increasing its viscosity. Another study by Backes et al. [29], investigated the printability, bioactivity, and biocompatibility of HA-PLA and TCP–PLA composites that were developed by twin-screw extruder with 5 and 10 wt%. The results showed good rheological behavior for both composites suitable for 3D printing using FDM. Subsequently, scaffolds were printed using neat PLA and its composites. The composite scaffolds formed calcium phosphate after soaking in SBF for 7 days, indicating excellent bioactivity. The compatibility results showed a higher cell attachment and proliferation for HA-PLA and TCP–PLA with 10 wt% when compared to the composites with 5 wt%. Also, HA-PLA and TCP–PLA with 10 wt% showed significantly higher DNA compared to neat PLA and the composites with 5 wt%. These studies showed that the increase of TCP content in the PLA matrix increases bioactivity and biocompatibility in vitro. Thus, it is worthwhile to develop TCP–PLA biocomposite-based filaments and utilize them in an FDM setup to develop design-specific TCP–PLA implants efficiently. However, FDM of filament-based composites requires careful processing, especially when developing mechanically robust implants.

It is well known that FDM-based printers are relatively easy to use yet utilizing them to process composite filaments requires careful processing. Usually, FDM printers have one or more nozzles and a printing heating bed. There are two main motors in the printer. The first motor controls the movement of the nozzle in X and Y directions over the printing bed. The second controls the movement of the printing bed up and down in the Z direction. FDM is a process in which a constant diameter filament of a thermoplastic polymer or its composite is fed into the nozzle where it melts and is extruded then deposited on the printing bed. The printing bed lowers after each entirely constructed layer by a distance equal to the layer height. This allows the next layer to get extruded and bonded to the previous one [30]. Notably, the material used in the FDM process must have specific rheological properties that allow it to get extruded through the nozzle without clogging it then be deposited on the bed and retain its shape. During the FDM process, the filament experiences a high shear rate at the nozzle while experiencing a low shear rate when deposited on the bed [19]. Therefore, the 3D printed filaments should be characterized by a shear-thinning behavior in which the material undergoes a significant reduction in viscosity at high shear rates. Viscoelasticity is another critical property that allows the 3D printed material to retain its shape once deposited on the printing bed [19,31]. The rheological performance of the polymer melt and mechanical properties of the printed structure get affected mainly by the nozzle temperature during the FDM process [32,33]. A higher nozzle temperature reduces the polymer melt viscosity, thus improving the polymer melt flow and deposition on the printing bed. Better flow and deposition of polymer melt enhances the bonding between the layer of the printed structure, thus, higher mechanical properties [33,34,35]. Moreover, the nozzle temperature directly affects the degree of crystallinity of the printed structure, affecting its mechanical properties [36,37,38].

Considering the importance of developing good-quality 3D printable filaments and optimization of FDM parameters to develop mechanically robust composite parts, in this work, TCP–PLA composite filament with 15 vol% TCP was first developed by a one-time process method. The 15 vol% of TCP has been chosen based on our previous study developing PLA composite [19]. The results of that study suggested that significant agglomeration can occur above 15 vol% ceramic content, especially if a single-screw extruder is used. A physical characterization was performed for the composite to ensure the existence and homogenous dispersion of TCP particles within the PLA matrix. Rheological tests were performed on pure PLA and TCP–PLA composite at temperatures 190 °C, 200 °C, 210 °C, and 220 °C. The temperature range was chosen depending on the manufacturer’s suggested printing temperature of PLA. The filaments were then used to 3D print dog-bone shaped bars for tensile testing as per ASTM standards. Finally, the relationship between the rheological and mechanical properties of the 3D printed TCP–PLA composites was investigated.

## 2. Materials and Methods

### 2.1. Materials

Polylactic acid (PLA) was purchased in a pellet form from 3DXTECH (Grand Rapids, MI, USA). The β-tricalcium phosphate (nanoXIM-TCP200) was purchased from Fluidnova (Maia, Portugal). βTCP (Ca_3_(PO_4_)_2_) has a purity of ≥95%, bulk density of 0.35 (±0.1) g/cm^3^, and a particle size (d_50_) of 5 (±2) µm.

### 2.2. Fabrication of 3D Printable βTCP/PLA Composite Filaments

**Figure 1:**
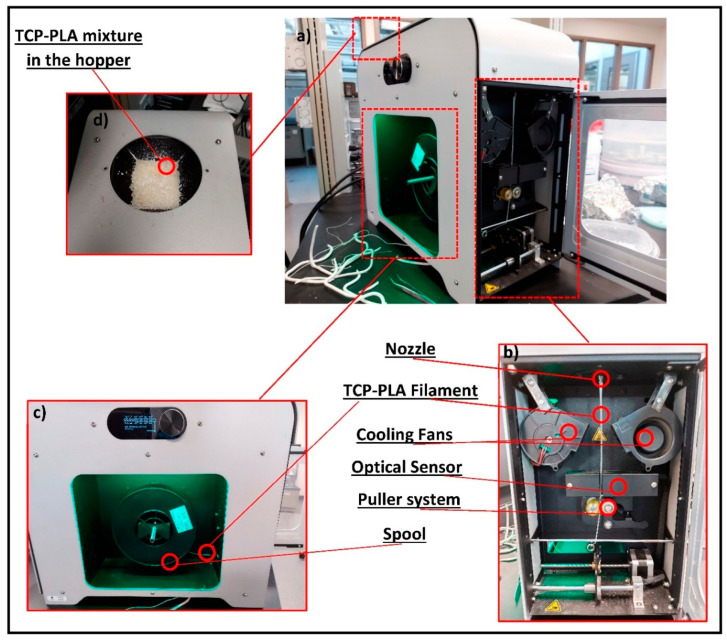
Fabrication process of 3D printable TCP–PLA composite filament. (**a**) 3Devo filament maker extruder. (**b**) Front view of the extruder showing the filament extrusion out of the nozzle to the puller system. (**c**) Side view of the extruder showing the spooling process of the filament. (**d**) Top view showing the TCP powder with the PLA pellets in the hopper.

The PLA pellets were dried for 4 h at 60 °C. Blair Tornado II paint mixer (Swartz Creek, MI, USA) was used to mix the PLA pellets with TCP powder (15 vol% βTCP) for 15 min. The 3D printable TCP–PLA filament with a constant diameter of 1.75 mm was produced using filament maker extruder device from 3devo (Atoomweg, The Netherlands). The 3devo filament maker extruder is equipped with a single screw and two fans at the exit of the nozzle to air quench the filaments. The filament maker extruder has four heaters where heater 1 is close to the nozzle and heater 4 is at the feeding zone. The heaters’ (1 through 4) temperature is kept at 185 °C, 190 °C, 190 °C, and 185 °C, respectively. The βTCP–PLA mixture was fed to the feeding zone when all the four heaters reached the setpoints. Then the fan speed was set to 85% and the screw speed was set to 5 rpm. Once a filament started coming out of the nozzle, it was guided to the spool through the positioner to form constant diameter filaments, as shown in Figure 1.

### 2.3. Physical Characterization

The physical characteristics of the TCP–PLA filament were investigated as follows. The distribution of TCP particles in the PLA matrix and the morphological features of the TCP–PLA filament were investigated using a scanning electron microscope (SEM) (S4800, Hitachi, Tokyo, Japan) equipped with an energy dispersion X-ray spectroscopic detector (EDX). The specimens were prepared for SEM imaging by coating with gold utilizing a sputter coater (108auto, Cressington, UK). The functional groups of the filament were characterized using Fourier Transform Infrared spectroscope (FTIR) (UMA-600 Microscope, Varian Excalibur Series). X-ray Diffraction (XRD) with monochromated Cu Kα radiation (Rigaku Ultima III, Woodlands, TX, USA) was used to evaluate the crystallographic structure of TCP, PLA, and TCP–PLA composite. The XRD was operated at a voltage of 40 kV and a current of 44 mA for 2θ ranging from 10° to 45°, with a step size of 0.04 and scan speed of 1°/min. TCP diffraction peaks confirmed with ICDD 09-0432. The thermal properties of TCP, PLA, and TCP–PLA composite were investigated using Differential Scanning Calorimetry (DSC) (DSC 250, TA instruments, New Castle, DE, USA). The degree of crystallinity (X_cw_%) was calculated using Equation (1)

(1) $$\mathrm{Xcw}\left(\%\right)=\frac{H_{m}}{w_{f}\times H_{c}}\times 100\%$$

where H_m_ is the melt enthalpy, w_f_ is the weight fraction of PLA, and H_c_ is the melt enthalpy of 100% crystalline PLA and is equal to 93 J/g [39].

### 2.4. Rheological Properties

RDA III parallel plate rheometer with a plate of 25 mm in diameter (Rheometric Scientific, USA) was used to measure the rheological properties of pure PLA and TCP–PLA composite melts. A dynamic amplitude sweep test was conducted at 220 °C to evaluate the viscoelastic region for both PLA and TCP–PLA. Subsequently, a dynamic frequency sweep test was performed with 10% strain amplitude at temperatures of 190 °C, 200 °C, 210 °C, and 220 °C and the elastic (storage) modulus (G′), viscous (loss) modulus (G″), and complex viscosity (η*) were measured over a frequency range of 10–500 rad/s. Additionally, a steady state flow test was performed at 220 °C to measure the shear viscosity over a shear rate of 0.1–10 s^−1^ then compare it to the complex viscosity to investigate the applicability of the Cox–Merz rule. All rheological analyses were performed with a 1.2 mm gap between the parallel plates and under nitrogen environment.

### 2.5. 3D Printing

**Figure 2:**
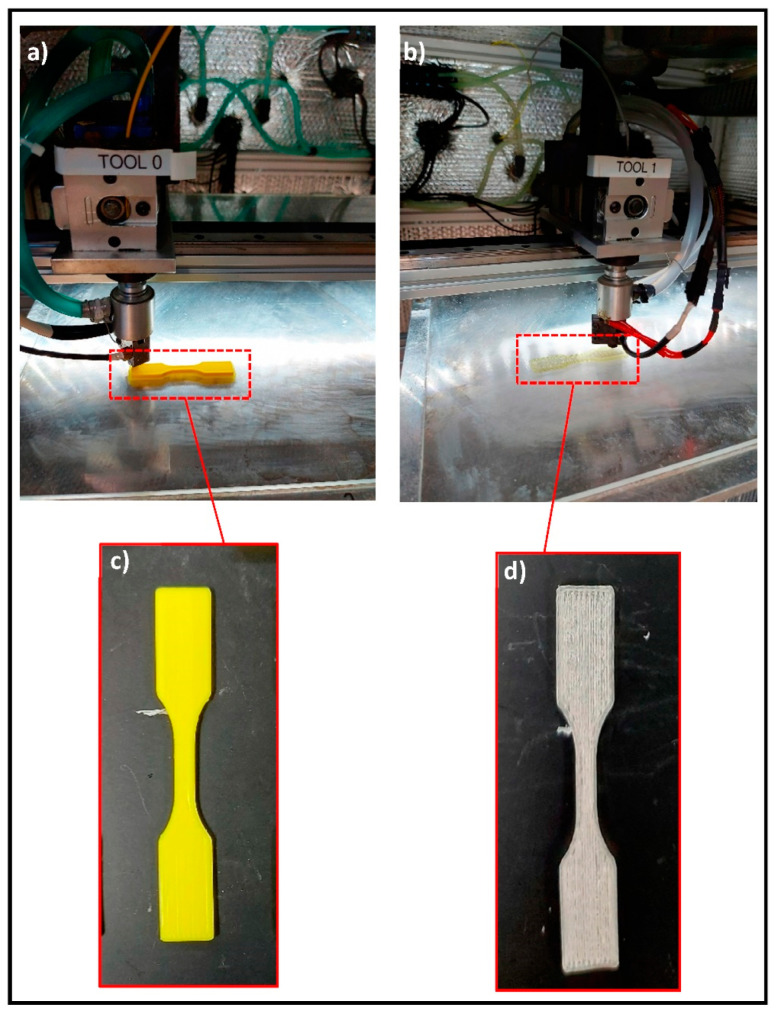
FDM process of tensile bars of (**a**) PLA and (**b**) TCP–PLA. The printed tensile bars of (**c**) PLA and (**d**) TCP–PLA.

FDM industrial grade 3D printer (AON M1, Montreal, Canada) was used in the printing processes where Simplify 3D^TM^ software was used to control the parameters of the printing process. PLA and TCP–PLA filaments were dried for 4 h at 60 °C. Subsequently, they were inserted into the printer’s extrusion nozzle with 0.6 mm diameter. The base plate temperature was maintained at 60 °C. However, different temperatures were used for the nozzle temperature. All the tensile bars were printed with a 100% infill and 0.3 mm layer height, as shown in Figure 2.

### 2.6. Mechanical Testing

PLA and TCP–PLA tensile bars type V were printed in accordance with ASTM D638. Type V tensile bar has a length of 63.5 mm, a grip section width of 9.53 mm, and a thickness of 3.4 mm. Instron Universal Tester (Norwood, MA, USA) with a 2 kN load cell and at a strain rate of 5 mm/min at room temperature was used to evaluate the tensile strength and Young’s modulus of the 3D printed PLA and TCP–PLA tensile bars. Bluehill software^®^ (Instron, Norwood, MA, USA) was used to collect and analyze the data.

### 2.7. Statistical Analysis

All the tests were conducted in triplicate and reported as mean ± standard deviation. One-way ANOVA followed by T-test was used to analyze the results’ significance and *p*-values less than 0.05 were considered statistically significant.

## 3. Results

**Figure 3:**
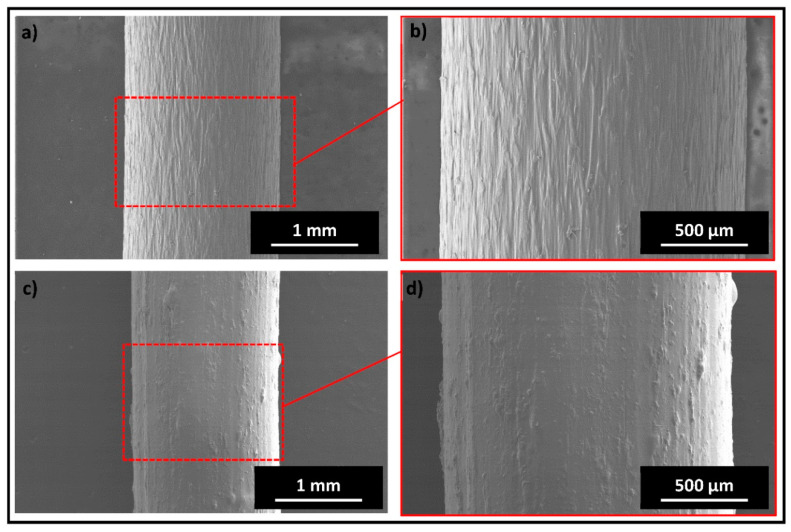
SEM images of PLA filament at (**a**) low and (**b**) high magnification and TCP–PLA filament at (**c**) low and (**d**) high magnification. (The scale bar represents the actual length).

**Figure 4:**
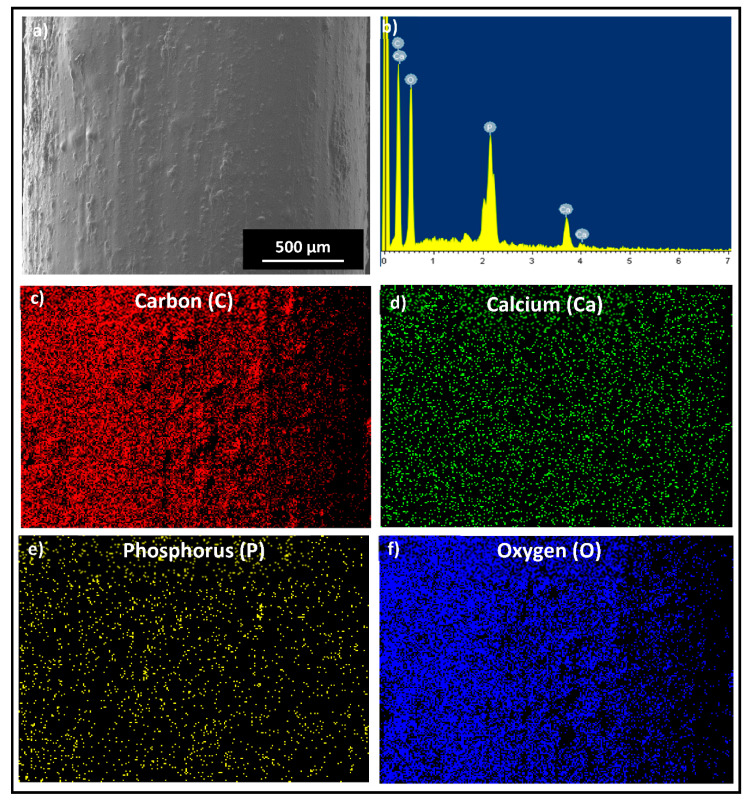
(**a**)SEM high magnification image of TCP–PLA filament. (**b**)EDX analysis of TCP–PLA filament. EDX element mapping of (**c**) Carbon, (**d**) Calcium, (**e**) Phosphorus, and (**f**) Oxygen. (The scale bar represents the actual length).

All the physical characterization results confirm the presence of TCP particles embedded in the PLA matrix. Figure 3 shows SEM images of PLA and TCP–PLA composite filaments. The micrographs confirmed the uniform distribution of TCP particles in the PLA matrix. Moreover, the SEM images show that TCP–PLA composite filament has a constant diameter similar to the PLA filament. Figure 4 shows the EDX and element mapping. The EDX picked a strong signal of carbon that corresponded to the PLA backbone. Also, EDX picked up peaks of TCP constituents, i.e., calcium (Ca), oxygen (O), and phosphorous (P). The element mapping results were in accordance with the EDX results. The element mapping signals also confirmed a homogenous distribution of all TCP constituents through the PLA matrix.

**Figure 5:**
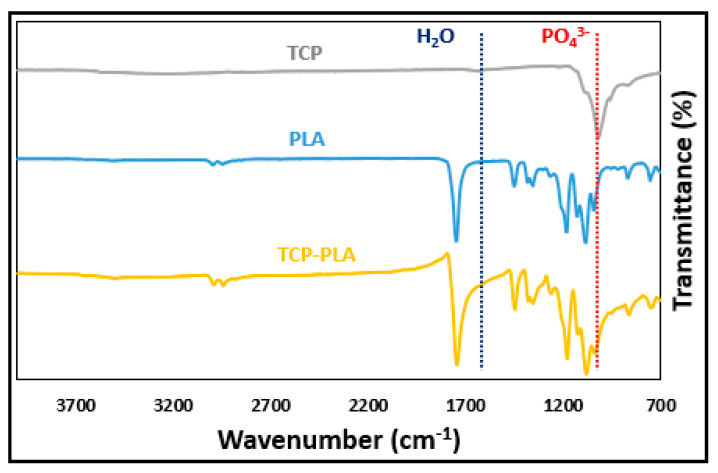
FTIR data of TCP, PLA, and TCP–PLA.

Figure 5 shows the FTIR data for TCP, PLA, and TCP–PLA composite filament. In the TCP spectrum, absorption bands were observed at 1022 and 1627 cm^−1^, corresponding to v3 phosphate ion (PO_4_^3−^) and OH^−^ functional group from the water molecule (H_2_O), respectively. In the PLA spectrum, bands were observed at 1061, 1747, and 2995 cm^−1^, which corresponds to C–O, C=O, and C–H stretching, respectively [40]. In the case of TCP–PLA composite, the typical PLA bands were observed as well as the PO_4_^3−^ and H_2_O absorption bands which confirmed the presence of TCP particles in the PLA matrix.

**Figure 6:**
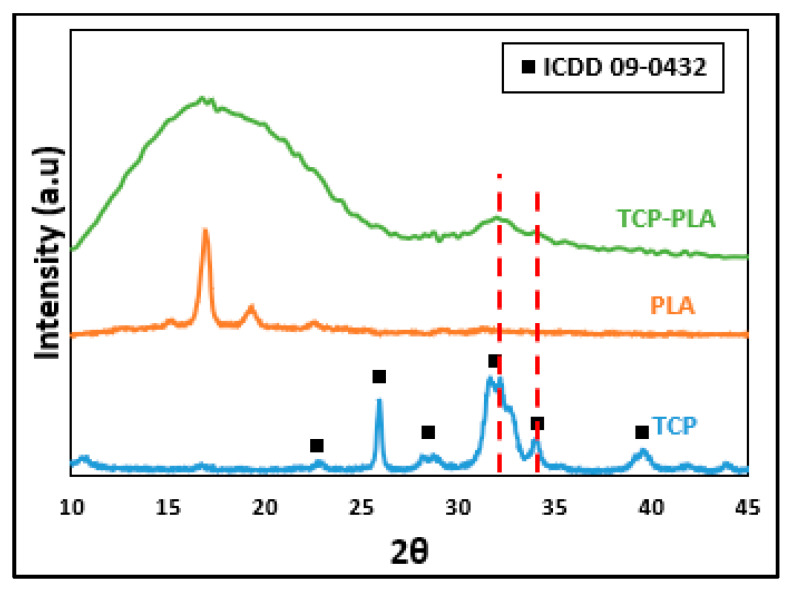
XRD patterns of TCP, PLA, and TCP–PLA.

Figure 6 shows XRD patterns of TCP, PLA, and TCP–PLA. The TCP diffraction pattern indicates the highly crystalline structure of the TCP powder, and no second phase was detected other than the βTCP. The PLA diffraction pattern detected a strong peak at 2θ of 17.2, revealing the highly crystalline structure of PLA. In the case of TCP–PLA, the diffraction pattern showed a broad hump from 2θ of 10 to 25 without any sharp peaks, which indicates a significant reduction in PLA crystallinity after processing the TCP–PLA composite. Moreover, the TCP–PLA diffraction pattern detected the two main sharp peaks of TCP at 2θ of 32 and 34, which indicates the presence of TCP in the TCP–PLA composite.

**Figure 7:**
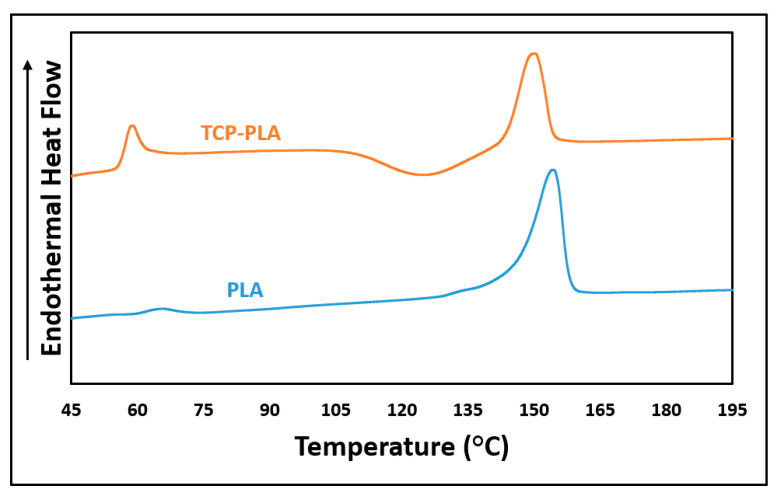
DSC results of PLA and TCP–PLA filaments.

**Figure 8:**
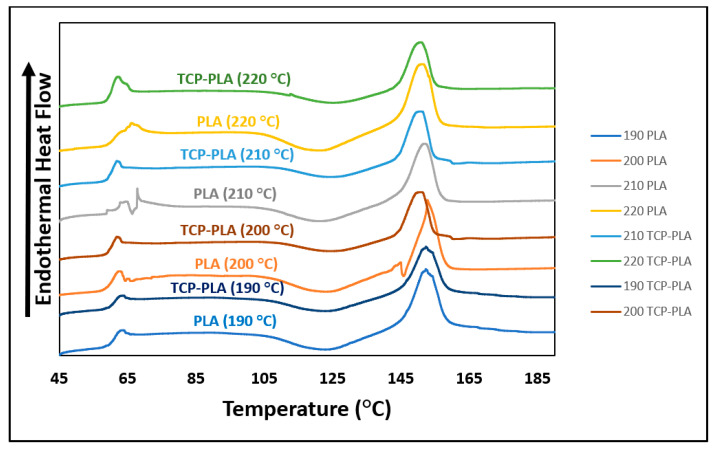
DSC results of PLA and TCP–PLA samples printed at different nozzle temperatures.

**Figure 9:**
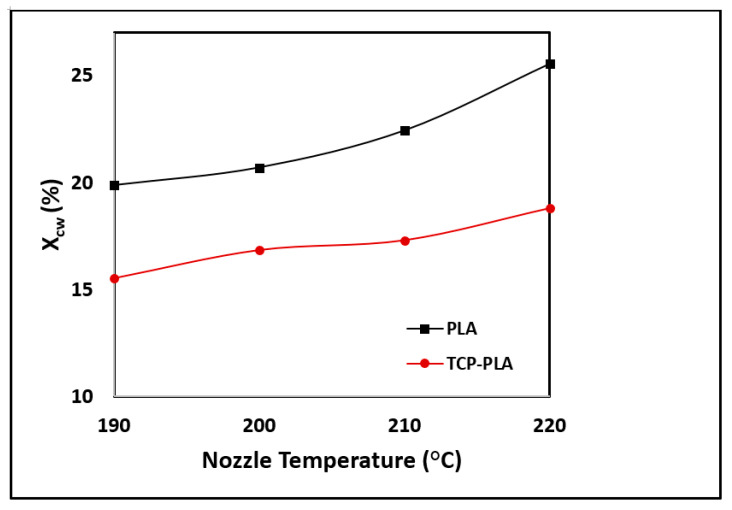
Crystallinity of PLA and TCP–PLA samples printed at different nozzle temperature (190 °C, 200 °C, 210 °C, and 220 °C).

Figure 7 shows the DSC results of PLA and TCP–PLA, and Table 1 summarizes their thermal properties including glass transition temperature (T_g_), crystallization temperature (T_c_), melting temperature (T_m_), enthalpy (H_m_), and crystallization percent (X_cw_). A slight reduction in the glass transition and melting temperatures was observed with the incorporation of TCP particles into the PLA matrix. Specifically, the T_g_ and T_m_ of TCP–PLA were lower than PLA by 13.07 °C and 3.87 °C, respectively. Moreover, the TCP particles had a significant effect in reducing the melting enthalpy. The melting enthalpy of TCP–PLA composite is lower than the PLA by 44.42% resulting in a substantial reduction in the TCP–PLA crystallinity. The reduction in thermal properties of TCP–PLA composite can be attributed to the decrease in crystallinity due to processing conditions and the disturbance of the PLA crystals by TCP particles. Figure 8 shows the DSC of the printed PLA and TCP–PLA at different nozzle temperatures, and Table 2 shows their thermal properties and crystallinity. The melting enthalpy increases with the nozzle temperature, resulting in increased crystallinity in the printed samples Figure 9.

**Figure 10:**
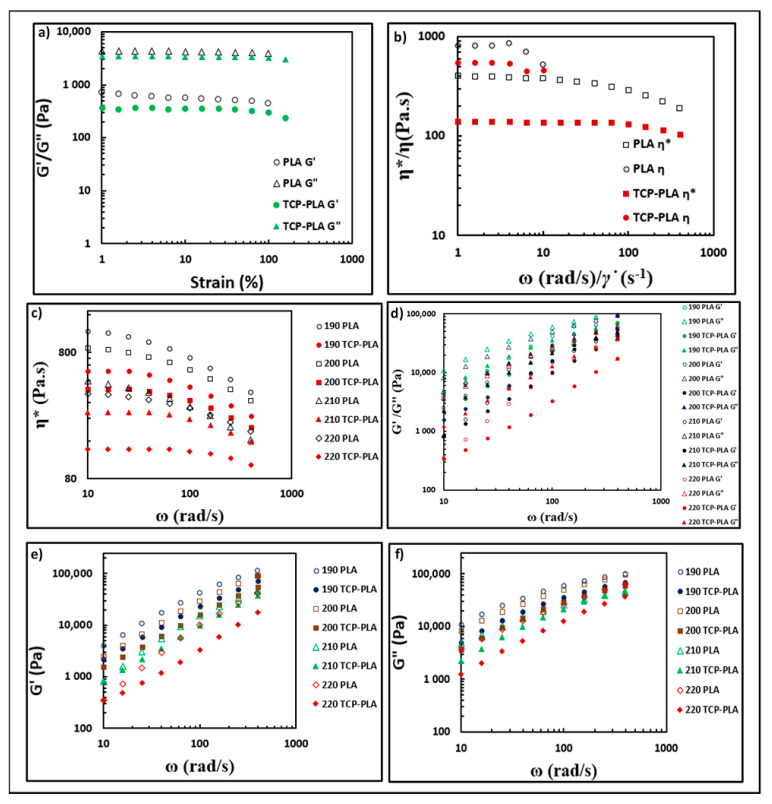
Rheological properties. (**a**) Dynamic strain sweep test (elastic modulus (G′)/viscous modulus (G″) vs. strain%). (**b**) Investigating Cox–Merz rule (complex viscosity (η*)/shear viscosity (η) vs. frequency (ω)/shear rate ($\dot{\gamma }$)). (**c**–**f**) Complex viscosity, elastic modulus, and viscous modulus obtained from the dynamic frequency sweep test.

Figure 10 shows the rheological properties of PLA and TCP–PLA. Figure 10a shows the dynamic sweep strain test where the G′ and G″ plotted versus the strain%. The G′ and G″ plateau represents the linear viscoelastic region of the melts. The G′ of PLA and TCP–PLA experienced a plateau from 1% to 100% strain value. The G″ of PLA and TCP–PLA experienced a plateau up to a strain value of 60%. Therefore, a strain of 10% was chosen for the constant strain dynamic frequency sweep test. Figure 10b shows the comparison between the dynamic shear rate sweep test and dynamic frequency sweep test, where the shear viscosity is a function of shear rate, and the complex viscosity is a function of frequency. That comparison indicated the failure of Cox–Merz rule for PLA and TCP–PLA as the shear viscosity did not match the complex viscosity at the range of shear rate/frequency from 1 to 10 Hz/ω. Figure 10c shows the complex viscosity of PLA and TCP–PLA obtained from the dynamic frequency sweep test at different nozzle temperatures. The PLA and TCP–PLA showed a shear thinning behavior at all temperatures as the complex viscosity at all runs reduced to lower values at high frequencies. The complex viscosity of all samples decreased with the increase of nozzle temperature. Moreover, the complex viscosity of TCP–PLA samples were less than those of PLA over the entire frequency range at all nozzle temperatures. Figure 10d shows the G′ and G″ of PLA and TCP–PLA as a function of frequency. The G″ was greater than G′ for PLA and TCP–PLA at all temperatures. Additionally, the viscoelastic moduli of samples at all temperatures increased with frequency increase.

**Figure 11:**
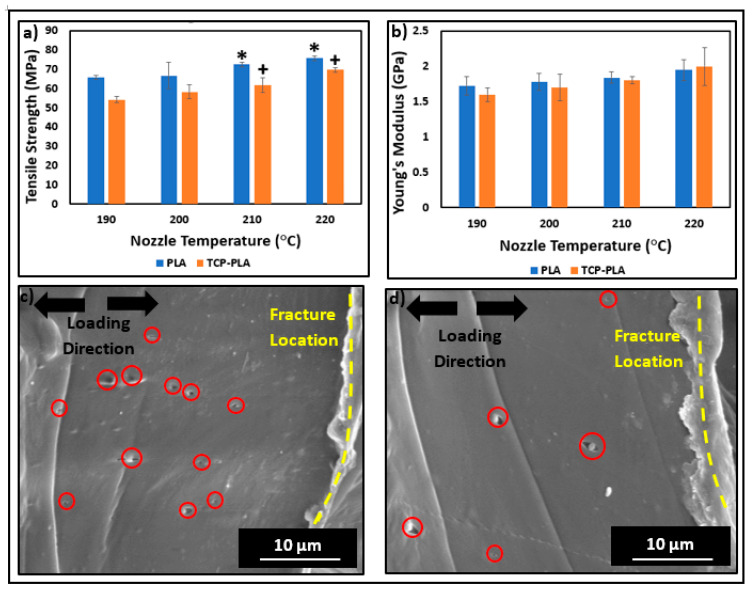
(**a**) Tensile strength and (**b**) Young’s modulus of PLA and TCP–PLA samples printed at different nozzle temperatures. (**c**) and (**d**) SEM images of tensile fractured surface morphology of TCP–PLA (Red circles locate the voids generated by TCP particles during tension). (The asterisk (*) and plus symbol are significant with respect to PLA and TCP–PLA samples printed at 190 °C, respectively). (The scale bar represents the actual length).

Figure 11 shows the mechanical properties of 3D printed PLA and TCP–PLA tensile bars at different nozzle temperatures. The results revealed a directly proportional relationship between the nozzle temperature and the mechanical properties. The PLA and TCP–PLA samples printed at nozzle temperature of 190 °C showed the lowest tensile strength with 65.8 (±0.971) MPa and 54.344 (±3.796) MPa, respectively, as shown in Figure 11a. In comparison, PLA and TCP–PLA samples printed at nozzle temperature of 220 °C showed a higher tensile strength with 75.77 (±1.147) MPa and 69.711(±6.23) MPa, respectively, as shown in Figure 11a. Similarly, Young’s modulus was lowest at nozzle temperature 190 °C with values of 1.725 (±0.134) GPa and 1.685 (±0.143) GPa for PLA and TCP–PLA, respectively, as shown in Figure 11b, and was highest at nozzle temperature of 220 °C with values of 1.837 (±0.156) and 2.161 (±0.332) for PLA and TCP–PLA, respectively. The PLA showed higher tensile strength at all nozzle temperatures as compared to the TCP–PLA, which can be explained by two reasons. First, the crystallinity of PLA samples was higher than TCP–PLA samples at all nozzle temperatures, as shown in Table 2 and Figure 9. Second, the voids generated by the delamination of TCP particles from the PLA matrix, shown in Figure 11c,d.

## 4. Discussion

In this study, a 3D printable TCP–PLA composite filament was developed using an effective one-step processing method that was first introduced in developing a PLA composite that incorporates amorphous magnesium phosphate (AMP) [19]. The results emphasized the effectiveness of that method in producing high-quality 3D printable filament composite characterized by a constant diameter filament with a homogeneous distribution of TCP particles in the PLA matrix, which is confirmed by the physical characterization of TCP–PLA composite filament. The SEM micrographs in Figure 3 showed the homogenous distribution of TCP particles in PLA matrix. Consistent with the SEM images, the EDX observations, presented in Figure 4, verified the same results with the homogenous distribution of all the TCP elements, including calcium, phosphorus, and oxygen in the PLA matrix, Moreover, the FTIR and XRD patterns have confirmed the existence of TCP particles in the developed TCP–PLA composite filament.

Notably, the XRD showed a reduction in TCP–PLA crystallinity post-processing. The DSC confirmed the reduction in TCP–PLA crystallinity by 13.38% compared to the PLA, Table 1. The reduction in composite crystallinity is due to the rapid cooling of the extruded filament by the two fans located at the exit of the nozzle, as shown in Figure 1. Rapid cooling and, thus, solidification of the TCP–PLA is required for the puller system to pull the filament and direct it to the spool, as shown in Figure 1b,c. Similar results were demonstrated in our previous study while developing a 3D printable AMP–PLA composite, where the crystallinity of the composite decreased by 12.56% compared to the PLA [19]. A study by Corcione et al. focused on developing a PLA-HA composite filament for FDM where PLA and HA powders were used to process PLA and PLA-HA composite filaments with different weight percent of HA by melt extrusion. The XRD patterns of the processed PLA and PLA-HA showed amorphous structure similar to the present study [41].

Rheological properties are critical in evaluating any FDM process, especially in comparing the behavior of two materials as in the present study. The rheological responses are important because the main stage in the FDM process is through the nozzle, where the filament melts then deposits and solidifies on the printing bed. The rheological analysis is usually used to estimate polymers’ and composites’ internal structure and predict their printability behavior. Moreover, it is the primary method to manage the reproducibility and preserve the quality of the 3D printed structures. It is required for any material used in FDM process to perform a shear thinning behavior. There is no reference for a specific reduction value in viscosity at higher shear rates that can confirm a successful FDM process. Therefore, the best method to predict a successful FDM process for new material is to compare with the rheological responses of a successfully 3D printed material via FDM.

The rheological properties of the PLA and TCP–PLA composite were investigated at different temperatures that correspond to the nozzle temperature at the actual FDM process. The complex viscosity values along the frequency range were lower at higher nozzle temperatures, indicating better material flowability through the nozzle. For instance, at a frequency of 10 rad/s, the complex viscosity of PLA and TCP–PLA at 220 °C are lower than those at 190 °C by 67.73% and 76.37%, respectively. At a frequency of 100 rad/s, the complex viscosity of PLA and TCP–PLA at 220 °C are lower than those at 190 °C by 60.2% and 70.53%, respectively. TCP–PLA samples showed lower complex viscosity values than PLA samples at all frequencies at the same nozzle temperature. For instance, at a nozzle temperature of 200 °C and frequency of 10 rad/s, the complex viscosity of TCP–PLA is lower than PLA by 56.4%. Similarly, at nozzle temperature of 210 °C and frequency of 400 rad/s, the complex viscosity of TCP–PLA is lower than the PLA by 49.69%. The good flowability associated with lower complex viscosity is favorable for the FDM process unless the material experiences an uncontrollable flow. In addition, lower viscosity reduces the probability of nozzle clogging and improves the material deposition, thus, better material bonding the previously printed layer [42,43].

The elastic and viscous moduli of TCP–PLA are lower than PLA at all the temperatures along with the frequency range, which indicates the lower mechanical properties of TCP–PLA as compared to the PLA. The G″ is higher than G′ at all temperatures and frequencies, confirming the PLA and TCP–PLA melt state and the printed structure’s ability to retain its shape [19,44]. The higher G′ of PLA compared to TCP–PLA at the same temperature can be explained by the strong interactions between the PLA chains that were disturbed by the TCP particles in the TCP–PLA composite [45]. Lower G′ means less rigidity which is more favorable in the FDM process as the material will move easily through the nozzle to the printing bed [46]. The present study investigated the applicability of the Cox–Merz rule, which is an empirical rule correlating the rheological properties obtained from dynamic frequency sweep test and steady-state test, η($\dot{\gamma }$) = η(ω), where $\dot{\gamma }$ = ω [47]. In other words, the Cox–Merz rule compares the shear viscosity as a function of shear rate to the complex viscosity as a function of frequency. The Cox–Merz rule has failed for both PLA and TCPP-PLA, as shown in Figure 10. Similar results were shown in a previous study while developing an AMP–PLA composite, where the Cox–Merz rule failed for PLA and the AMP–PLA composite [19]. Cicala et al. investigated three commercial types of PLA filament, including black, white, and green PLA filaments. In that study, the Cox–Merz rule failed for the black and white PLA filaments while applied for the green one [31]. Also, several studies in the literature found that the Cox–Merz rule is not applicable for some polymers, especially high molecular weight and functionalized polymers [48]. The importance of the Cox–Merz rule is due to its ability to increase the shear rate range that can be tested from the oscillatory rheometer since viscosity as a function of shear rate is hard to measure at a high shear rate due to significant sample deformation and secondary flows [48,49]. Thus, it helps in minimizing the number of samples and experiments needed to investigate rheological properties.

The nozzle temperature plays a critical role in the mechanical properties of the 3D printed structure [32,50]. Specifically, the nozzle temperature affects the rheology of the polymer melts through the nozzle—as discussed—and the crystallinity of the 3D printed structure, which are the main parameters affecting the mechanical properties of the 3D printed structure. Additionally, the nozzle temperature can affect the properties of a fully 3D printed layer while printing the other subsequent layers. Vanaei et al. investigated the temperature profile of 3D printed PLA structures by FDM [51]. According to that study, a specific deposited layer during FDM process can be affected by the nozzle temperature while depositing the five subsequent layers. In that study, K-type thermocouples [52] were used to monitor the layers’ cooling and reheating during the FDM process. The temperature profile of layer five was recorded and it showed that layer five first cooled down when it was entirely deposited, then it reheated while depositing layer six, then cooled again after the fully deposition of layer six, then reheated while depositing layer seven. In that study, layer five experienced four cooling reheating cycles that can directly affect its bonding and crystallinity. Like most other polymers, PLA is characterized by a semi-crystalline structure that contains amorphous and crystalline phases [53]. On the one hand, the amorphous phase is characterized by a random and intertwined structure of the polymer chains. On the other hand, the crystalline phase is characterized by a close polymer chains alignment with higher molecular order and stronger intermolecular forces. Therefore, the crystalline/amorphous ratio directly affects the density and mechanical properties of PLA [38,54,55]. In the present study, all the PLA and TCP–PLA printed samples at different nozzle temperatures exhibited a semi-crystalline structure, as shown in Table 2. However, the crystallinity of PLA and TCP–PLA printed samples increased with increasing the nozzle temperature, as shown in Figure 9. Also, that explains the increase in mechanical properties of PLA and TCP–PLA samples printed at higher nozzle temperature, as shown in Figure 11a,b. The present study agreed with the literature on the higher mechanical properties of PLA printed structures associated with higher nozzle temperature. Behzadnasab et al. studied the effect of the nozzle temperature on the physical and mechanical properties of printed PLA structures [56,57]. In that study, the PLA filament was developed in-house using a single screw extruder. The filament was then used to 3D print tensile bars at different nozzle temperatures ranging from 180 °C to 240 °C with a 20 °C gradient. The results confirmed the directly proportional relationship between the nozzle temperature and tensile properties. An increase in nozzle temperature from 180 °C to 240 °C increased the tensile strength from 34 MPa to 56 MPa [56]. Akhoundi et al. investigated the effect of nozzle temperature, and heat treatment (annealing) after printing on the mechanical properties of 3D printed PLA structures [58]. In that study, two groups of PLA were printed at different nozzle temperatures of 210 °C, 220 °C, 230 °C, 240 °C, and 250 °C. The first group was tested for their tensile mechanical properties without heat treatment, while the second group was annealed for one hour at 110 °C. The mechanical properties of both groups increased with increasing the nozzle temperature. An increase in nozzle temperature from 210 °C to 250 °C resulted in an increase in tensile strength from 60 MPa to 65.75 MPa and a tensile modulus from 3 GPa to 4.9 GPa. The second group (annealed) exhibited higher mechanical properties than the first group (non-annealed) at all nozzle temperatures due to the increase in crystallization of annealed samples. The second group’s maximum tensile strength and tensile modulus were 67.4 MPa and 5.65 GPa, respectively [58]. El Magri et al. studied the influence of nozzle temperature and infill orientation on the mechanical properties of PLA and carbon fiber (CF)-PLA [59]. In the first part of that study, tensile bars of PLA and CF-PLA were printed at nozzle temperatures ranging from 190 °C to 240 °C with a 10 °C gradient with fixed infill orientation. The results showed an increase in tensile strength and Young’s modulus of PLA and CF-PLA with increasing nozzle temperatures up to 230 °C, after which the mechanical properties were decreased due to PLA degradation.

## 5. Conclusions

A one-step process method was successfully utilized to develop 3D printable TCP–PLA composite filaments. The TCP–PLA composite filament exhibited a constant diameter and a homogenous distribution of TCP particles within the polymer matrix. The TCP–PLA filaments were successfully used to print tensile bars with different nozzle temperatures in an FDM setup. We observed that a higher nozzle temperature helped enhance the tensile strength of the printed parts, with the highest values recorded for parts printed at a nozzle temperature of 220 °C. However, TCP–PLA printed samples experienced lower mechanical properties than PLA at all nozzle temperatures because of the micropores generated by TCP particles. Notably, higher nozzle temperatures also increased the crystallinity of both the PLA and TCP–PLA printed parts, which is considered the primary reason for the mechanical property enhancement. The rheological investigation showed an improvement of rheological properties with increasing nozzle temperature. PLA and TCP–PLA mechanical properties increased with increasing the nozzle temperature. Overall, our study confirmed the successful development of 3D printable TCP–PLA filaments and utilized them effectively to fabricate design-specific parts with favorable mechanical properties that greatly benefit orthopedic and dental biomedical applications.

## Figures and Tables

**Table 1:** Thermal properties of PLA and TCP–PLA filaments obtained from DSC.

Specimen	T_g_ (°C)	T_c_ (°C)	T_m_ (°C)	H_m_ (J/g)	X_cw_ (%)
PLA	70.07	-	154.26	30.439	32.73
TCP–PLA	57	125.05	150.39	16.919	19.354

**Table 2:** Thermal properties of PLA and TCP–PLA samples printed at different nozzle temperatures.

Specimen	Nozzle Temperature (°C)	T_g_ (°C)	T_c_ (°C)	T_m_ (°C)	H_m_ (J/g)	X_cw_ (%)
PLA	190	61.58	122.86	152.3	18.47	19.86
	200	65.8	122.98	152.8	19.242	20.69
	210	68.63	121.28	151.82	20.864	22.434
	220	70.2	121.14	151.29	23.771	25.56
TCP–PLA	190	60.52	125.1	150.85	13.704	15.51
	200	60.19	124.39	151.01	14.87	16.83
	210	60.68	125.05	151.1	15.28	17.29
	220	62.92	124.58	150.67	16.603	18.792

## Data Availability

Not applicable.
